# Prof. T. Romeyn Beck

**Published:** 1848-07

**Authors:** 


*Prof. T. Romeyn Beck.*—From a paragraph in the Albany Evening Journal, we learn that Prof. Beck has resigned his connection with the Albany Academy, and designs to devote his attention to a speedy revisal of his work on the "*Elements of Medical Jurisprudence,*" the last edition of which (the seventh) has been for two years out of print.

